# Child feeding knowledge and practices among women participating in growth monitoring and promotion in Accra, Ghana

**DOI:** 10.1186/1471-2393-14-180

**Published:** 2014-05-29

**Authors:** Sandra Gyampoh, Gloria Ethel Otoo, Richmond Nii Okai Aryeetey

**Affiliations:** 1 Department of Nutrition and Food Science, University of Ghana, Legon, P.O. Box LG 134, Accra, Ghana; 2 School of Public Health, University of Ghana, Legon, P.O. Box LG 13, Accra, Ghana

**Keywords:** Growth monitoring and promotion, Child welfare clinic, Child feeding, Complementary feeding, Breastfeeding

## Abstract

**Background:**

Child undernutrition and poor feeding practices remain a concern in Ghana. The Growth Monitoring and Promotion (GMP) programme seeks to empower mothers to provide appropriate child care. Although the program has been implemented in Ghana for over four decades, little is known about its impact on child feeding outcomes. The current study assessed the association between GMP exposure and mothers' child feeding knowledge and practices in the Accra Metropolitan Area (AMA), Ghana.

**Methods:**

A cross-sectional survey of 199 mother-child pairs accessing child welfare services in six public health facilities in the AMA was conducted. A structured questionnaire was used to collect data on respondent characteristics and child feeding knowledge; a 24-hour dietary recall tool was used to record child feeding practices. Linear regression analysis was used to determine the association between mothers' exposure to GMP and their knowledge and practices on child feeding.

**Results:**

Seventy four percent of mothers had not missed any scheduled child welfare clinic sessions. Over 60% of mothers knew the appropriate age of introduction of foods; 86% also gave correct response regarding minimum number of times their child should be fed daily. About 81% of children less than 6 months were exclusively breastfed in the preceding 24 hours, although 36% had received water since birth. Forty two percent of children 6–23 months received dietary diverse meals while 64% were fed the required number of times in a day. Overall, only 32% of children 6–23 months received a minimum acceptable diet in the preceding 24 hours. A higher GMP exposure was positively associated with feeding knowledge scores among mothers with children below 6 months (*p* < 0.05).

**Conclusion:**

Although most mothers were knowledgeable about recommendations, feeding practices were suboptimal, especially complementary feeding. GMP exposure was associated with feeding knowledge only among mothers with children less than 6 months. Strengthening of feeding counselling focused on children above 6 months is recommended.

## Background

An estimated 35% of global under-five deaths, and 50-70% of diarrhoeal diseases, measles, malaria and lower respiratory infections in developing countries are attributable to child undernutrition [1,2]. Undernourished children are prone to poor mental, physical and physiological development, and are at increased risk of infections and death due to nutrient deficiencies [2,3]. In Ghana, the latest Multiple Indicator Cluster Survey [4] shows that about 13% of children below age 5 years are underweight, 23% are stunted, and 6% are wasted. In the Greater Accra Region (GAR), underweight is found among 8.3% of children 0-5 years while 13.7% and 5.4% are stunted and wasted respectively [4].

Undernutrition is often common in poor-resource countries where appropriate breastfeeding and complementary feeding (CF) practices are suboptimal [5-8]. Usually, complementary foods are introduced too early and are often of poor quality and quantity in terms of nutrient diversity, density and feeding frequency [5,9]. In Ghana, only 46% of children below six months are exclusively breastfed. In the GAR of Ghana, only 21.1% of children between 0-5 months were exclusively breastfed, being the lowest rate recorded in the country. Often, the diet of Ghanaian children is mainly made of grains, roots and tubers [4,10]. Improving child feeding practices among mothers therefore remains essential to improving child nutrition and survival in Ghana.

The Ghana Health Service's Child Welfare Clinic (CWC) is a comprehensive child health service that includes immunization, nutrient supplementation, and growth monitoring and promotion. The Growth Monitoring and Promotion (GMP) component of the CWC is focused on empowering mothers to know about and become competent to practice appropriate child care, feeding, and health seeking. These outcomes are pursued using individualized and group counselling [11,12]. The GMP provides an opportunity for interaction between public health workers and mothers regarding the health and wellbeing of their children [13].

Although the GMP program has been implemented in Ghana for over four decades [14], little is known about its impact on child feeding outcomes. A previous study in Ghana reported on mothers' comprehension of growth charts used as part of the GMP to monitor child growth patterns as well as factors influencing mother's attendance to CWC [14]. The current study assessed the association between GMP exposure and mothers' knowledge and practices on the feeding of their young children under two years in the Accra Metropolitan Area of Ghana.

## Methods

### Study sites

The study was carried out in six public health facilities, one in each of the six health sub-metropolitan areas in the Accra Metropolitan Area (AMA) of Ghana. The AMA is the most densely populated administrative district in the Greater Accra region with an estimated 4.5 million people living in the capital city of Accra. The Metropolitan area is sub-divided into six sub metros: Kpeshie, Osu-Clottey, Okaikoi, Ayawaso, Ablekuma and Ashiedu-Keteke.

### Study design and participants

The study used a cross-sectional design and surveyed 199 mother-child pairs accessing CWC services in the selected health facilities. This sample size was adequate to detect a 15% difference in prevalence of maternal knowledge across regular and non-regular CWC attendees at a power of 80% and confidence level of 95% [15].

One health facility was randomly selected from a list of public-managed health facilities in each sub-metropolitan area. The number of mother-child pairs recruited from each health facility was proportional to the number of children below 24 months enrolled in the particular facility as at June 2011. Data for the study were collected between November 2011 and January 2012.

The study protocol was approved by the Institutional Review Board of the Noguchi Memorial Institute for Medical Research (NMIMR-IRB), University of Ghana. The AMA as well as the selected facilities provided documented approval to facilitate work at each selected study site. Mother-child pairs attending monthly CWC sessions were approached and recruited into the study after informed consent was obtained between November 2011 and February 2012. Inclusion criteria were mothers with children aged 0–23 months, of normal birth weight (≥2.5 kg), of singleton birth and with no obvious signs of illness.

### Study questionnaire

#### Background characteristics

A structured questionnaire was used to record data on socio-demographic characteristics of mother-child pairs and child feeding knowledge and practices of mothers. The number of CWC attended was obtained from child health records. Interviews were conducted individually with the assistance of a trained field assistant.

### Child feeding knowledge

To assess mothers' child feeding knowledge, a questionnaire previously used in Haiti was adapted [16] based on recommended feeding practices [17]. Questions covered the appropriate age of introduction of water and other liquids, grains/roots and tubers, vegetables, fruits, dairy products, eggs and flesh foods. For mothers with children 6–23 months, the appropriate feeding frequency for their child's age and the recommended duration of continued breastfeeding were included.

### Child feeding practices

Feeding practices were assessed using a single 24-hour dietary recall of foods the child was fed in the 24 hours preceding the interview. Scores developed from the 24 hour recall were adapted from the same study conducted in Haiti [16] and based on feeding recommendations [17]. Variables for assessing feeding practices among mothers with children less than 6 months were whether the child received breast milk, formula, or semi-solid/solid foods in the preceding 24 hours. Among mothers with children 6-23 months, data on dietary diversity, feeding frequency and if the child received breast milk were obtained from the 24 hour recall.

### Exposure to GMP

Mothers' exposure to GMP was assessed by how often they visited CWC. The number of months a mother had brought her child to the clinic in relation to the scheduled visits per the age of the child was obtained from the child’s health records.

### Data analysis

Data were entered and analyzed using SPSS, version 16.0. Means, standard deviation, frequencies and percentages were used to describe data.

To calculate total SES scores, individual scores ranging from 0.5 to 2.5 were given for the types of tenancy and the main sources of cooking energy. A score of 1 was given for the possession of each electrical appliance. Child feeding knowledge scores were determined by assigning a score of 1 for knowing the appropriate age of introduction of each food and appropriate feeding frequency for child's age. Scores for knowledge of the recommended duration of continued breastfeeding ranged from −2 (not knowing or indicating ≤ 6 months) to 2 (indicating 6 to 24 months or beyond). Variables used in child feeding practice scores among mothers with children less than 6 months were whether or not a child had been fed breast milk, formula, or semi-solid/solid foods in the preceding 24 hours. A score of 1 was assigned for giving breast milk, not feeding formula and not feeding semi-solid/solid foods. Among mothers with children 6-23 months, a score of 1 was assigned for each food group fed, for feeding the recommended frequency or more and for breastfeeding.

The ratio of a child's age to the number of CWC visits attended was calculated and used in the linear regression analysis to determine the association between mother's exposure to GMP and their child feeding knowledge and practices. The proportion of age of child to number of scheduled clinics attended, the SES score, child feeding knowledge and practice scores and relevant background characteristics were included in the model.

## Results

### Background characteristics of participants

Of the 199 children included in the study, 98 were males and 101 were females; with a mean age of 6.78 ± 4.65 completed months (Table 1). The mean age for mothers was 28.09 ± 5.31 completed years. Most mothers had secondary level education or higher (77.4%) and only few did not have any form of employment (17.1%). Close to half of mothers (48.7%) lived in a rented house and more than half used Liquefied Petroleum Gas (LPG) as their main source of energy for cooking (65.3%). Table 1 shows the health worker as the main source of child feeding information for most mothers (58.3%) while family and friends were indicated as a source by a little over a quarter of mothers (26.1%).

**Table 1:** Background characteristics of mother-child pairs (N = 199)

**Characteristic**	**n (%)/Mean ± SD**
***Sex of child***	
Male	98 (49.2)
Female	101 (50.8)
***Age***	
Age of child in completed months (mean and SD)^a^	6.78 ± 4.65
Age of mother in completed years (mean and SD)^a^	28.09 ± 5.31
***Marital status of mother***	
Single/widowed	46 (23.1)
Married	153 (76.9)
***Level of education of mother***	
Primary/less^b^	45 (22.6)
Secondary/higher	154 (77.4)
***Employment status of mother***	
Unemployed	34 (17.1)
Trader	81 (40.7)
Artisan	61 (30.7)
Domestic worker^c^	4 (2.0)
Professional^d^	19 (9.5)
***Type of accommodation***	
Caretaker	14 (7.0)
Company/government	5 (2.5)
Family house	45 (22.6)
Rented	97 (48.7)
Own	38 (19.1)
***Main source of cooking energy***	
Firewood	1 (0.5)
Charcoal	66 (33.2)
Kerosene	1 (0.5)
LPG	130 (65.3)
Electric cooker	1 (0.5)
***Household Items***	
Radio	175 (87.9)
Television	183 (92.0)
Refrigerator	157 (78.9)
Personal Computer	59 (29.6)
***Source of child feeding information***	
Health worker at CWC	116 (58.3)
Family and friends	52 (26.1)
Self-application	26 (13.1)
Information in child health records book	3 (1.5)
Media sources (TV/radio/print/internet)	2 (1.0)

^a^SD, standard deviation ^b^Less than completed Junior secondary school education ^c^House cleaner/cook. ^d^institutional employees including nurses, teachers, secretaries, etc.

### Attendance to CWC

Table 2 shows the attendance of mothers to CWC. Seventy four percent of mothers had not missed any scheduled CWC sessions. Among those who had missed one or more scheduled CWC sessions, the highest percentage (46.2%) were mothers with children aged 6–8 months, while the lowest percentage, 13.5%, were mothers with children 0–5 months.

**Table 2:** CWC attendance among mothers (N = 199)

***Attendance***	***n (%)*/Mean ± SD**
Mean attendance	6.09 ± 4.3
Missed no CWC sessions	147 (73.9)
Missed one or more CWC sessions	52 (26.1)
***Distribution among missed attendance***	
Age of child (months)	
0-5	7 (13.5)
6-8	24 (46.2)
9-11	11 (21.2)
12-23	10 (19.2)

### IYCF knowledge and practices

Majority of all mothers were able to indicate the appropriate age of introduction of foods and most of those with children 6 months or older were also able to indicate the minimum number of times their child should be fed in a day (Table 3). However about a quarter of mothers indicated that vegetables, eggs and flesh foods (meat/fish/poultry/organ meats) be introduced after 8 months, while 17.1% indicated that water/other liquids be given before the child was 6 months old (Table 3).

**Table 3:** Mothers’ knowledge of age of appropriate introduction of foods and feeding recommendations among children 0-23 months (N = 199)

	**Age indicated (months)**			
**Foods**	***<6***	***6-8***^***a***^	***>8***	***Not sure***
	***n (%)***	***n (%)***	***n (%)***	***n (%)***
Water/other liquids	34 (17.1)	163 (81.9)	2 (1.0)	0 (0.0)
Staples (grains, roots & tubers)	13 (6.5)	174 (87.4)	10 (5)	2 (1.0)
Vegetables	8 (4.0)	136 (68.3)	45 (22.6)	10 (5.0)
Fruits	10 (5.0)	153 (76.9)	30 (15.1)	6 (3.0)
Dairy products	11 (5.5)	154 (74.9)	27 (13.6)	12 (6.0)
Eggs (yolk & whole)	4 (2.0)	141 (70.9)	50 (25.1)	4 (2.0)
Flesh foods (meat/fish/poultry/organ meats)	4 (2.0)	133 (66.8)	56 (28.1)	6 (3.0)
Knows minimum number of times to feed child in a day^†^^b^ n (%)	95 (86.4)			
Knows recommended duration of continued breastfeeding^†^^c^ n (%)	62 (56.4)			

^†^Values for these variables are for mothers with children ≥6 months. ^a^Recommended age for introduction of foods in addition to breast milk [17]. ^b^Twice/more for breastfed infants 6–8 months, 3 times/more for breastfed children 9–23 months and 4 times/more for non-breastfed children 6–23 months [17]. ^c^≥ 24 months [17].

Table 4 shows the child feeding practices of all mothers. Thirty six percent of mothers with children less than 6 months had given water to their child since birth, although a greater percentage (80.9%) had exclusively breastfed the child in the preceding 24 hours. Among children 6 months or older, 65.5% were given water at 6 months while 20.9% were introduced to complementary foods before 6 months. Recommended feeding practices were better practiced among mothers with children below 6 months than those of older children. More than half of children above 6 months received meals from fewer than four food groups, indicating low dietary diversity. Overall, only about 32% of children aged 6–23 months received a minimum acceptable diet.

**Table 4:** Child feeding behaviour reported by mothers of children 0–23 months (N = 199)

**Feeding**	**n (%)**
***Children <6 months (n = 89)***	
Introduction of water (since birth)	
Started	32 (36.0)
Not yet	57 (64.0)
Feeding practices in the preceding 24 hours	
Exclusive breastfeeding	72 (80.9)
Breast milk and formula	5 (5.6)
Breast milk and complementary foods	6 (6.7)
Not receiving any breast milk	6 (6.7)
***Children ≥6 months (n = 110)***	
Age of introduction of water	
<6 months	38 (34.5)
At 6 months	72 (65.5)
Age of introduction of complementary foods	
<6 months	23 (20.9)
At 6 months	87 (79.1)
Feeding practices in the preceding 24 hours	
Fed grains, roots and tubers	100 (90.9)
Fed legumes and nuts	23 (20.9)
Fed dairy products	53 (48.2)
Fed flesh foods (meat/fish/poultry/organ meats)	44 (40.0)
Fed eggs	8 (7.3)
Fed vitamin A rich fruits and vegetables	36 (32.7)
Fed other fruits and vegetables	58 (52.7)
Met dietary diversity/more^a^	46 (41.8)
Met adequate feeding frequency^b^	70 (63.8)
Met minimum acceptable diet^c^	35 (31.8)
Breastfed	100 (90.9)

^a^Consumption of meals containing 4 or more food groups (PAHO/WHO, [17].

^b^2 times/more for breastfed infants 6–8 months, 3 times/more for breastfed children 9–23 months and 4 times/more for non-breastfed children 6–23 months [17].

^c^Fed foods meeting the recommended minimum dietary diversity and the minimum feeding frequency [17].

### Association between GMP exposure and child feeding knowledge and practices

In linear regression analysis shown in Table 5, feeding knowledge score was positively associated with both GMP exposure and age of mother, if child was below 6 months (*p* < 0.05). Feeding practice score was also found to be significantly associated with age of the child among these mothers but not with GMP exposure. The feeding knowledge scores of mothers with children 6-23 months were not significantly associated with any of the variables in the model. In terms of feeding practices among these mothers, feeding knowledge scores were positively associated with feeding practice scores (*p* < 0.01) but was not found to have a significant relationship with GMP (Table 6).

**Table 5:** Factors associated with child feeding knowledge among mothers (N = 199)

**Variable**	**Regression coefficient**	**P-value**	**R**^**2**^
***Mothers with children <6 months (n = 89)***			
Feeding knowledge score			
Age of mother	.250	.019	.069
Level of education of mother	.061	.556	
Age of child/number attended (proportion)	.225	.034	
***Mothers with children 6-23 months (n = 110)***			
Feeding knowledge score			
Age of mother	.132	.172	.000*
Level of education of mother	.076	.434	
Age of child/number attended (proportion)	.043	.656	

*The model was not found to be significant.

**Table 6:** Factors associated with child feeding practices among mothers (N = 199)

**Variable**	**Regression coefficient**	**P-value**	**R**^**2**^
***Mothers with children <6 months (n = 89)***			
Feeding practice score			
Age of mother	.099	.354	.130
Age of child	-.352	.001	
Level of education of mother	-.071	.540	
Socioeconomic score	-.065	.576	
Feeding knowledge	.172	.105	
Age of child/number attended (proportion)	.033	.764	
***Mothers with children 6-23 month (n = 110)***			
Feeding practice score			
Age of mother	-.114	.242	.089
Age of child	.179	.069	
Level of education of mother	.132	.174	
Socioeconomic score	.011	.911	
Feeding knowledge	.273	.004	
Age of child/number attended (proportion)	.066	.479	

## Discussion

The study assessed the feeding knowledge and practices among mothers participating in GMP and also investigated the association between GMP exposure and mothers' child feeding knowledge and practices. In the study, a majority of mothers had not missed any CWC sessions. However among mothers who had missed one or more sessions, the highest percentage (46.2%) was found among those with children 6-8 months. The appropriate age for introducing other foods to children was known by majority of mothers, although about a quarter indicated wrong timing for introduction of vegetables, eggs and flesh foods. Feeding practices in the preceding 24 hours was appropriate among children 0-5 months compared to children 6–23 months. Exclusive breastfeeding (EBF) was practiced by 80.1% of mothers with children 0-5 months in the preceding 24 hours. In children 6-23 months over half of them did not receive a minimum acceptable diet in the previous day. GMP exposure was found to be associated with feeding knowledge of mothers with children 0-5 months old.

The ability of mothers to practice recommended feeding practices has been associated with maternal nutrition knowledge [18,19]. Thus it might be expected that with over 80% of mothers in this study indicating the appropriate age of introduction of water/other liquids, a similar trend will be seen in the practice of EBF. However, having knowledge alone may not always result in best practices [8]. Similar to results found by previous studies in Nigeria and Ghana [19-21], a contradiction was observed between mothers' knowledge and practice of EBF. The results from this study show that fewer mothers practiced EBF from birth, as indicated by the introduction of water/other liquids, than was shown by the 24 hour recall and this is comparable to findings in a study on EBF in Ghana [20]. This contrast between knowledge and practices may be attributed to barriers such as maternal employment, maternal health, cultural beliefs and practices and social pressure from family and friends [13,19,22-24].

The transition from EBF to CF is associated with several challenges in developing countries, including infrequent feeding, low energy and less nutrient dense foods, poor food storage and sanitation and food taboos [9,22,23]. These constraints make the nutritional and energy requirements of the growing child difficult to meet, making undernutrition more likely during this period [25]. Generally, feeding practices among children less than 6 months were shown to be better in the preceding 24 hours than were found for CF practices. Most children were fed meals made from grains, roots and tubers than from other foods in the preceding 24 hours as found in the Ghana Demographic and Health Survey [10]. Comparable to the Multiple Indicator Cluster Survey in Ghana [4], more children aged 6–23 months were fed the minimum recommended times or more in a day than were fed dietary diverse diets, with few meeting the minimum acceptable diet. The implication of this is that, nutrient requirements may not have been met in over half of the children in this study. To ensure that complementary feeding practices are adequate for optimal child growth from 6 months, accurate and consistent information and skilled support are essential as are for EBF [1]. It has been said that *'Considerable global and national efforts and attention have been devoted to breastfeeding promotion to the neglect of complementary feeding practices'*[8].

As the results of this study have shown, health services remain a major source of child feeding information for mothers [13,26,27]. It is expected that, the consistent monthly interaction between mothers and health workers as part of the GMP will provide not only knowledge, but also the support mothers need to undertake recommended feeding practices. Mothers' exposure in GMP was positively associated with the feeding knowledge scores of mothers with children less than 6 months but not on their feeding practices. This emphasises the need for repeated supportive counselling rather than the relaying of only breastfeeding messages [8,20,28]. Exposure to GMP was not found to be associated with child feeding knowledge or practices among mothers with children 6 months or older. This may draw attention again to the less attention given to CF [8]. However for these mothers', knowledge of child feeding recommendations was seen to have a positive association with feeding practices. An implication that if much attention is given to complementary feeding counselling and support during GMP sessions an impact will be seen in feeding practices and subsequently on child nutritional status.

In areas where nutrition counselling which is age-appropriate and specific to the family environment has been offered through the health system, improvements in the knowledge of mothers and the diets of children have been observed [27-31]. Mothers find it difficult to practice what they are told when messages are non-specific with less attention to a mother's household condition or availability of foods [1,32]. However, most CWC visited during this study offered no or generic nutrition messages and even fewer were observed to have group counselling sessions conducted by a nutritionist or community health nurse on some of the clinic days. Such nutrition messages that are non-age specific and non-individualized during GMP have been observed in other developing countries [33-35]. These practices do not enable the GMP programme to effectively improve mothers' knowledge and practices for better child growth outcomes [11,12]. The poor delivery of counselling has been attributed to a lack of required knowledge and skills on the part of health workers, heavy demand relative to personnel, lack of incentives and motivation, inadequate supervision, uncooperative and mistrusting mothers [12,27,35,36].

### Study limitations

Results in this study may not be generalizable to other regions as the study was conducted only in the Greater Accra Region which may differ from other regions in socio-demographic characteristics of respondents and health facility characteristics. Also in assessing knowledge, caregivers may speculate correct answers which may show good scores but may not reflect reality.

## Conclusion

Results from this study indicate that although most mothers participating in GMP in the AMA of Ghana were knowledgeable about child feeding recommendations, feeding practices were suboptimal, especially among children receiving complementary feeding. GMP exposure was, however, not associated with child feeding knowledge and practices; an exception was the association between GMP exposure and practices of mothers with children under 6 months. For the GMP programme to realize its objectives of improving child growth through influencing care and feeding practices, the nutrition counselling and support, should be strengthened among mothers of children receiving complementary feeding.

## Competing interests

The authors declare that they have no competing interests.

## Authors’ contributions

SG conceived the study, participated in study design, prepared study tools, collected and analyzed data and prepared the manuscript. GEO supervised the study, participated in study design, finalizing study tools, data analysis and critically revising the manuscript. RA supervised the study, participated in study design, finalizing study tools, data analysis and critically revising the manuscript. All authors read and approved the final manuscript.

## Authors’ information

SG (MPhil) is a recent graduate of the Department of Nutrition and Food Science, University of Ghana, Legon. GEO (PhD) is a lecturer at the Department of Nutrition and Food Science at the University of Ghana, Legon. RA (MPH, PhD) is a Senior Lecturer at the Population, Family and Reproductive Health Department at the School of Public Health of the University of Ghana.

## Pre-publication history

The pre-publication history for this paper can be accessed here:

http://www.biomedcentral.com/1471-2393/14/180/prepub
